# Plasminogen activator inhibitor 1 is not a major causative factor for exacerbation in a mouse model of SARS-CoV-2 infection

**DOI:** 10.1038/s41598-023-30305-8

**Published:** 2023-02-22

**Authors:** Takashin Nakayama, Tatsuhiko Azegami, Maki Kiso, Masaki Imai, Ryuta Uraki, Kaori Hayashi, Akihito Hishikawa, Norifumi Yoshimoto, Ran Nakamichi, Erina Sugita-Nishimura, Eriko Yoshida-Hama, Yoshihiro Kawaoka, Hiroshi Itoh

**Affiliations:** 1 Department of Internal Medicine, Keio University School of Medicine, 35 Shinanomachi, Shinjuku-ku, Tokyo, 160-8582 Japan; 2 Keio University Health Center, 4-1-1 Hiyoshi, Kohoku-ku, Yokohama-shi, Kanagawa, 223-8521 Japan; 3 Division of Virology, Institute of Medical Science, University of Tokyo, Tokyo, 108-8639 Japan; 4 The Research Center for Global Viral Diseases, National Center for Global Health and Medicine Research Institute, Tokyo, 162-8655 Japan; 5 Influenza Research Institute, Department of Pathobiological Sciences, School of Veterinary Medicine, University of Wisconsin-Madison, Madison, WI USA

**Keywords:** Infectious diseases, Vaccines

## Abstract

Coronavirus disease (COVID-19) caused by severe acute respiratory syndrome coronavirus 2 (SARS-CoV-2) remains a global pandemic. Although several vaccines targeting SARS-CoV-2 spike proteins protect against COVID-19 infection, mutations affecting virus transmissibility and immune evasion potential have reduced their efficacy, leading to the need for a more efficient strategy. Available clinical evidence regarding COVID-19 suggests that endothelial dysfunction with thrombosis is a central pathogenesis of progression to systemic disease, in which overexpression of plasminogen activator inhibitor-1 (PAI-1) may be important. Here we developed a novel peptide vaccine against PAI-1 and evaluated its effect on lipopolysaccharide (LPS)-induced sepsis and SARS-CoV-2 infection in mice. Administration of LPS and mouse-adapted SARS-CoV-2 increased serum PAI-1 levels, although the latter showed smaller levels. In an LPS-induced sepsis model, mice immunized with PAI-1 vaccine showed reduced organ damage and microvascular thrombosis and improved survival compared with vehicle-treated mice. In plasma clot lysis assays, vaccination-induced serum IgG antibodies were fibrinolytic. However, in a SARS-CoV-2 infection model, survival and symptom severity (i.e., body weight reduction) did not differ between vaccine- and vehicle-treated groups. These results indicate that although PAI-1 may promote the severity of sepsis by increasing thrombus formation, it might not be a major contributor to COVID-19 exacerbation.

## Introduction

In late December 2019, severe acute respiratory syndrome coronavirus 2 (SARS-CoV-2) was identified as the causative agent for various atypical respiratory diseases^1^. Coronavirus disease (COVID-19) caused by SARS-CoV-2 is spreading exponentially around the world, and the World Health Organization declared COVID-19 as a pandemic on March 11, 2020^2^; COVID-19 has led to more than 500 million cases and 6.3 million deaths as of July 2022^3^. Although therapeutic agents such as dexamethasone and remdesivir are effective in improving various clinical outcomes of COVID-19, it is also important to fundamentally suppress the COVID-19 pandemic, and vaccinations have a central role in this^4–6^. Several vaccines targeting SARS-CoV-2 spike protein were rapidly developed and have shown outstanding effects in preventing the infection, onset, and exacerbation of COVID-19^6^. However, the emergence of mutations in the viral spike protein that increase viral transmissibility or decrease its antigenicity may reduce the efficacy of existing vaccines^7, 8^. In addition, the omicron variant, which carries many mutations in its spike protein, is able to evade most of the anti-SARS-CoV-2 neutralizing monoclonal antibodies currently available^9^.

Most patients with COVID-19 are asymptomatic or paucisymptomatic initially, but some of them develop both progressive respiratory failure and multiple organ failure^10, 11^. The mortality rate of COVID-19 is higher than for other viral pneumonias^12^. Although the details are unclear, the increased thrombosis associated with COVID-19 may contribute to its high mortality^13^. A COVID-19-mediated thromboinflammatory state associated with endotheliopathy can lead to pulmonary and systemic thrombosis through the enhanced release of plasminogen activator inhibitor-1 (PAI-1), von Willebrand factor, factor VIII, or angiopoietin-2 from endothelial cells; this mechanistic pathway is considered to be the possible major pathogenesis of organ injury in severe COVID-19^14–20^. Although the frequency of thrombosis in patients with COVID-19 admitted to intensive care units is approximately 40.0%, several autopsy studies indicate underrecognition of this symptom, and more patients may actually develop thrombosis than expected^17, 18, 21^.

In this context, PAI-1 has been proposed to play an important role in thrombus formation in patients with COVID-19^22–26^. Activation of the fibrinolytic system to dissolve fibrin is dependent on the conversion of plasminogen to plasmin by the physiologic activators of tissue type and urokinase type plasminogen activator (t-PA and u-PA). PAI-1 inhibits the formation of plasmin and subsequent fibrinolysis by inactivating both t-PA and u-PA^24^. Patients with sepsis typically show a marked increase in PAI-1, which often leads to organ dysfunction and disseminated intravascular coagulation, so the serum PAI-1 level is a significantly predictive biomarker of sepsis severity and mortality^25, 27^. In addition, increasing evidence indicates that, by promoting the accumulation of extracellular matrix, PAI-1 furthers the progression of lung fibrotic diseases including acute respiratory distress syndrome and idiopathic pulmonary fibrosis^28^. In COVID-19 cases, overproduction of proinflammatory cytokines or activation of the complement system can increase the circulating level of PAI-1^23, 25^. Several recent studies have reported that the level or activity of PAI-1 was elevated in critically ill patients with COVID-19 compared with healthy controls and correlated closely with the severity of COVID-19^25, 29–31^. Whether increased levels or activity of PAI-1 are a cause or consequence of COVID-19 exacerbation remains unclear; nevertheless inhibiting PAI-1 function is a potential strategy for preventing COVID-19 disease progression. Indeed, clinical trials of an oral small-molecule inhibitor of PAI-1 (TM5614) for patients with COVID-19 are underway worldwide (JRCT2021200018 and NCT04634799).

Not only oral medication but also vaccination could be useful approaches to inhibit PAI-1 activity. Vaccination offers the benefits of a long-lasting therapeutic effect and cost-effectiveness^32^. In addition, SARS-CoV-2 spike protein mutations might not hamper the efficacy of vaccines against the self-antigens involved in the pathogenesis of COVID-19 exacerbation. Here, we developed a novel peptide vaccine targeting PAI-1 to attenuate thrombus formation and prevent aggravation of COVID-19. First of all, to confirm that this vaccine properly functions, we evaluated whether PAI-1 vaccination decreased organ damage and mortality in mice with lipopolysaccharide (LPS)-induced sepsis, a model of systemic thromboinflammation where PAI-1 is a well-known exacerbating factor. We then evaluated the effect of our PAI-1 vaccine on thrombus formation in vivo and in vitro. Finally, we assessed whether our PAI-1 vaccine improved survival in a mouse model of severe SARS-CoV-2 infection.

## Results

### Antibody response to PAI-1 vaccine

**Figure 1:**
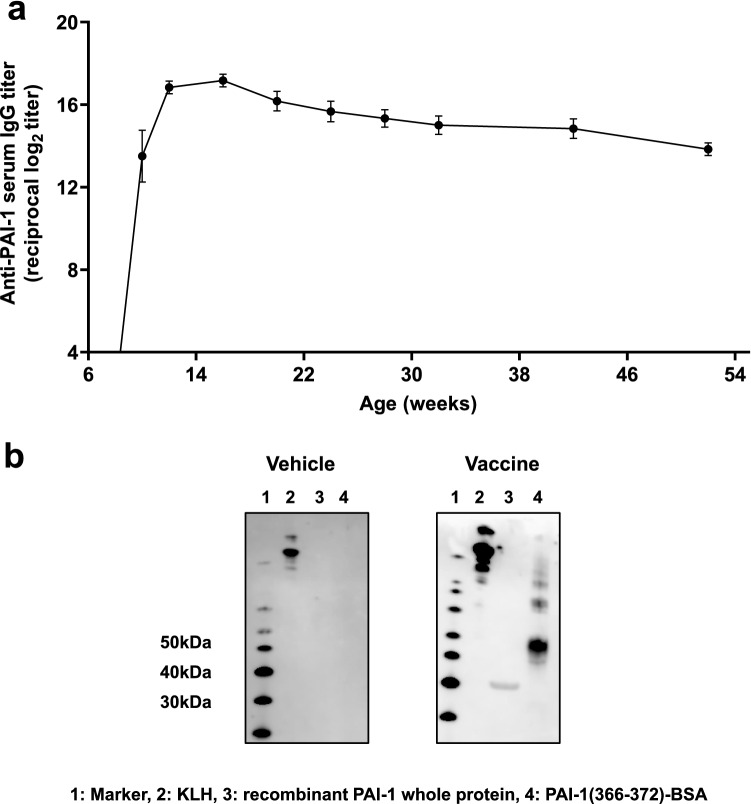
Evaluation of PAI-1 vaccine-elicited antibodies in mice. (**a**) Mice (*n* = 6 per group) received PAI-1 vaccine at 6, 8, and 10 weeks of age, after which serum samples were obtained at 4-week intervals from 12 to 52 weeks of age, and antigen-specific IgG antibody titers were evaluated by using ELISA. (**b**) Western blotting analyses evaluated whether IgG antibodies present in the serum of mice immunized with PAI-1 vaccine or KLH vehicle bound the vaccine antigen or recombinant whole PAI-1. Data are expressed as mean ± SEM (error bars).

As the epitope of our PAI-1 vaccine, we selected the amino acid sequence that is identical between humans and mice and contains the binding sites for t-PA and u-PA. We conjugated this PAI-1 partial peptide with keyhole limpet hemocyanin (KLH) and administered the product with Freund’s adjuvant subcutaneously to male C57BL/6J mice (*n* = 6) three times (i.e., at 6, 8, and 10 weeks of age), with a 2-week interval between immunizations (Fig. 1a). This protocol successfully elicited antigen-specific serum IgG antibody titers (reciprocal log_2_ titer 13.5 ± 3.1) at 2 weeks after the second immunization; these titers peaked (reciprocal log_2_ titer 17.2 ± 0.8) at 16 weeks of age and then gradually decreased (reciprocal log_2_ titer 13.8 ± 0.8) until 52 weeks of age. Furthermore, we evaluated the ability of PAI-1vaccine-elicited antibodies to bind to recombinant human PAI-1 (Fig. 1b). Unlike those of KLH vehicle–treated mice, serum IgG antibodies in the mice immunized with the PAI-1 vaccine recognized not only the epitope peptide but also recombinant PAI-1 whole protein.

### Effects of PAI-1 vaccine on organ function in an LPS-induced sepsis model

**Figure 2:**
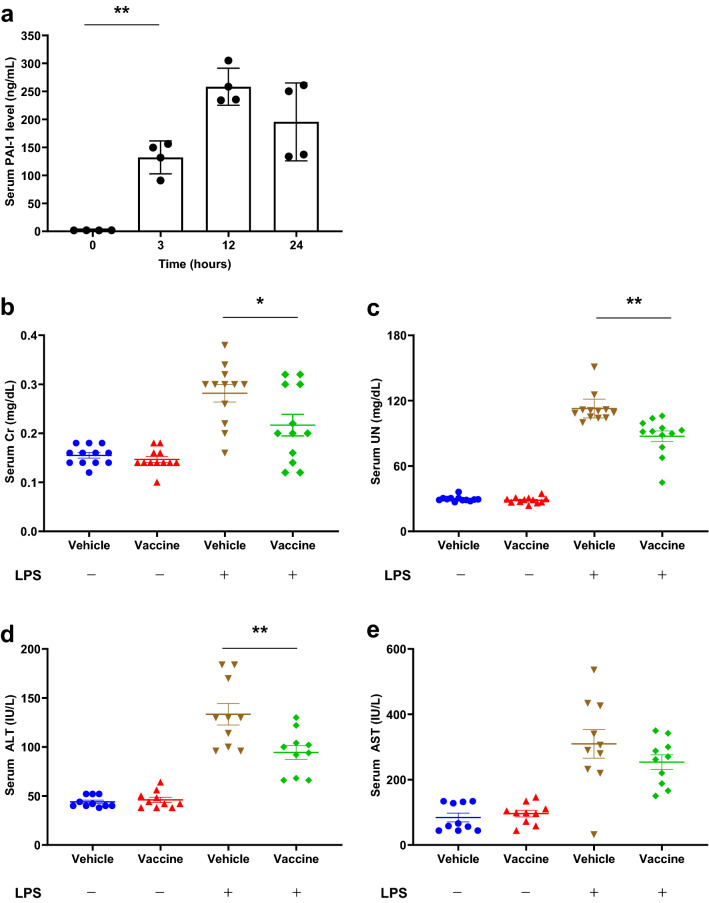
Effects of PAI-1 vaccine on organ function in a mouse model of LPS-induced sepsis. (**a**) Serum samples were obtained from non-immunized mice (*n* = 4 per group) before and at 3, 12, and 24 h after challenge with 6 mg/kg LPS, and serum PAI-1 levels were measured. (**b–e**) Mice (*n* = 8 per group) were immunized with the PAI-1 vaccine or KLH vehicle and then injected with or without 6 mg/kg LPS. Serum samples were obtained 24 h after LPS challenge, and creatinine (Cr), urinary nitrogen (UN), alanine aminotransferase (ALT), and asparagine aminotransferase (AST) levels were measured. Data are expressed as mean ± SEM (error bars). Significant differences in serum PAI-1 levels between before and 3 h after LPS administration, and in biomarkers of organ damage between the LPS-challenged vaccine and vehicle groups were determined by using Student’s *t*-test. **P < 0.01; *P < 0.05 compared with the value before LPS administration or for the LPS-challenged vehicle-treated group.

We first confirmed that the administration of LPS increased serum PAI-1 levels in mice. In preliminary investigations, we determined the 50% lethal dose (LD50) of LPS as approximately 12 mg/kg; we then administered LPS at a dose of 6 mg/kg (0.5 × LD50) to 13-week-old wild-type mice (*n* = 4). The serum PAI-1 level (baseline, 1.9 ± 0.1 ng/mL) increased dramatically beginning at 3 h after LPS administration (132.1 ± 29.3 ng/mL) (*P* < 0.01), peaked at 12 h (258.2 ± 33.1 ng/mL:), and then gradually decreased until 24 h (195.5 ± 69.6 ng/mL) (Fig. 2a).

To evaluate the effect of PAI-1 vaccine on organ damage in the LPS-induced sepsis model, we measured the serum biomarkers creatinine (Cr), urea nitrogen (UN), alanine aminotransferase (ALT), and aspartate aminotransferase (AST). Mice (*n* = 12 per group) were immunized three times with the PAI-1 vaccine (vaccine group) or KLH only (vehicle group) prior to LPS administration; LPS-untreated groups were established also. At 3 weeks after the last immunization, mice intraperitoneally received 6 mg/kg LPS (0.5 × LD50), and serum samples were collected at 24 h after LPS administration. Treatment with LPS increased serum levels of Cr, UN, ALT, and AST (Fig. 2b–e). However, the Cr and UN levels (which are markers of renal function) after LPS administration were lower in the vaccine group compared with the vehicle group (Cr: 0.22 ± 0.08 vs 0.28 ± 0.06 mg/dL, *P* < 0.05; UN: 87.3 ± 17.0 vs 112.7 ± 13.6 mg/dL, *P* < 0.01). Regarding liver function, PAI-1 vaccination decreased the serum ALT concentration and tended to affect AST levels (ALT: 94.4 ± 22.4 vs 133.4 ± 34.6 IU/L, *P* < 0.01; AST: 253.6 ± 70.4 vs 309.6 ± 138.9 IU/L, *P* = 0.27). These results indicate that the PAI-1 vaccine had a protective effect against LPS-induced organ injury in our mouse sepsis model.

### Effects of PAI-1 vaccine on survival in a mouse model of LPS-induced severe sepsis

**Figure 3:**
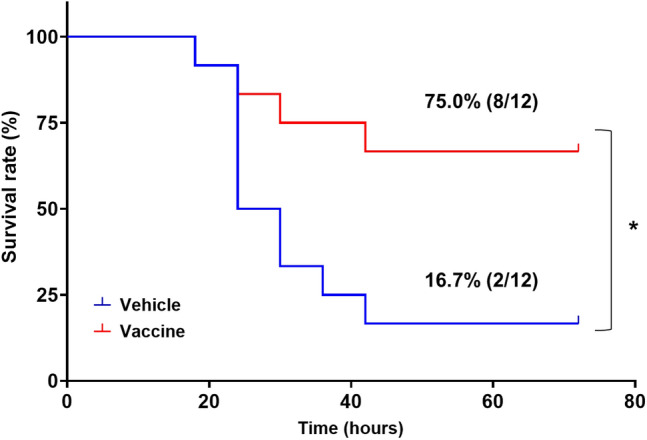
Effects of PAI-1 vaccine on survival rate in a mouse model of LPS-induced severe sepsis. Mice (n = 12 per group) immunized with PAI-1 vaccine or KLH vehicle received 15 mg/kg LPS and were subsequently monitored for survival for 72 h. Data are expressed as mean ± SEM (error bars). Survival was visualized by using the Kaplan–Meier method, and differences in survival were evaluated by using the log-rank test. *P < 0.05 compared with the value for the vehicle-treated group.

We then used our LPS-induced severe sepsis model to evaluate the effect of our PAI-1 vaccine on septic shock. Mice (*n* = 12 per group) were immunized three times with either the PAI-1 vaccine or KLH vehicle and were challenged with 15 mg/kg LPS (1.25 × LD50), after which mortality was observed for 72 h. The survival rate during the study period was 16.7% in vehicle group compared with 75.0% in the vaccine group (*P* < 0.05) (Fig. 3), thus indicating that PAI-1 vaccination significantly reduced mortality due to LPS-induced severe sepsis.

### Fibrinolytic functions of PAI-1 vaccine in LPS-induced sepsis model and plasma clot assay

**Figure 4:**
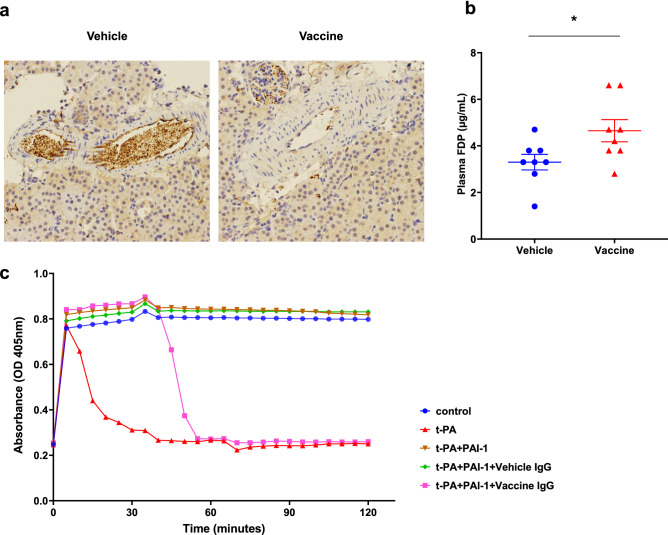
Fibrinolytic functions of PAI-1 vaccine in a mouse model of LPS-induced sepsis and plasma clot assay. Mice (*n* = 8 per group) were immunized with PAI-1 vaccine or KLH vehicle and challenged with 6 mg/kg LPS; kidney and plasma samples were obtained 24 h after LPS injection. (**a**) Kidney sections were stained with anti-fibrinogen antibody, and (**b**) plasma fibrin degradation products were measured. Data are expressed as mean ± SEM (error bars). Statistical difference between vaccine and vehicle groups with LPS challenge was determined by using Student’s *t*-test. *P < 0.05 compared with the value for the vehicle-treated group. (**c**) The following six arms were established in the plasma clot lysis assay: control (normal human plasma + assay buffer + human thrombin + CaCl_2_), control + t-PA, control + tPA + PAI-1, control + tPA + PAI-1 + KLH vehicle-elicited IgG, and control + tPA + PAI-1 + PAI-1 vaccine-elicited IgG. Clot lysis was observed for 120 min.

We evaluated the effects of PAI-1 vaccine on thrombus formation in LPS-induced sepsis model, in which suppressed-fibrinolytic-type disseminated intravascular coagulation leads to microvascular thrombosis and subsequently impaired organ function^33^. Preliminary investigations indicated that LPS formed more apparent thrombosis in renal arteries compared with the pulmonary or hepatic arteries. Therefore, we confirmed the fibrinolytic functions of PAI-1 vaccine with LPS-augmented thrombosis in small renal arteries. We intraperitoneally injected immunized and vehicle-treated mice with 6 mg/kg LPS (0.5 × LD50) and collected kidney and plasma samples at 24 h afterward, respectively. We then performed immunohistochemical staining with an anti-fibrin antibody to assess the degree of thrombosis. Whereas kidney samples from the vehicle group showed prominent thrombosis, vaccinated mice had less fibrin deposition in renal arteries (*n* = 3 mice per group) (Fig. 4a). In addition, plasma levels of fibrin degradation products (FDP) were higher in the vaccine group compared with vehicle group (4.7 ± 1.3 vs 3.3 ± 1.0 µg/mL; *P* < 0.05), suggesting that fibrinolytic activity was enhanced in the vaccinated mice (*n* = 8 per group) (Fig. 4b).

To examine fibrinolytic activity of PAI-1 vaccine-elicited IgG in vitro, we performed plasma clot lysis assays. In this assay, fresh-frozen plasma from healthy human donors immediately clotted when mixed with calcium chloride and thrombin, and the clots did not spontaneously dissolve during the 2-h observation period (Fig. 4c). Adding t-PA to the mixture of human plasma, calcium chloride, and thrombin induced clot lysis, but providing PAI-1 canceled out the fibrinolytic activity of tPA. However, including IgG obtained from PAI-1-vaccinated mice in the reaction mix induced clot lysis again, whereas that from mice immunized with KLH did not. These findings demonstrate that PAI-1 vaccine-elicited antibodies were able to accelerate the degradation of fibrin by inhibiting the antifibrinolytic function of PAI-1.

### Effects of PAI-1 vaccine on survival and symptomatology in a mouse model of SARS-CoV-2 infection

**Figure 5:**
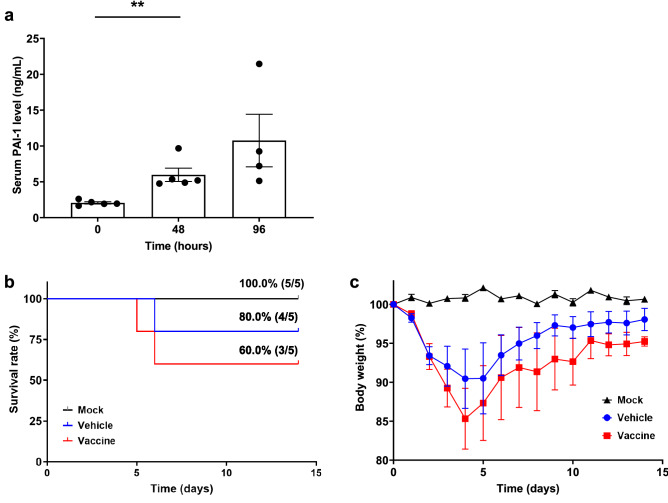
Effects of PAI-1 vaccine on survival and body weight change in a mouse model of SARS-CoV-2 infection. (**a**) Serum samples were obtained from non-immunized mice (*n* = 4 or 5 per group) before and at 48 and 96 h after intranasal infection with mouse-adapted SARS-CoV-2, and serum PAI-1 levels were measured. (**b**) The survival and (**c**) body weight of mock-infected mice and SARS-CoV-2-infected mice immunized with PAI-1 vaccine or KLH vehicle (n = 5 per group) were monitored for 14 days. Data are expressed as mean ± SEM (error bars). Significant differences in serum PAI-1 levels between before and 48 h after infection were determined by using Student’s *t*-test. **P < 0.01 compared with the values before infection. Survival was visualized by using the Kaplan–Meier method. Differences in survival were evaluated with the log-rank test; differences in body weight were determined by using a linear mixed-effects model.

Challenge with SARS-CoV-2 increased the serum PAI-1 level of mice^34^. In our mice (*n* = 4 or 5 per group), the serum PAI-1 level (baseline, 2.1 ± 0.4 ng/mL) significantly began to increase at 48 h after exposure to the virus (5.9 ± 2.1 ng/mL) (*P* < 0.01) and was even higher at 96 h afterward (10.8 ± 7.3 ng/mL) (Fig. 5a). Then, we evaluated the effect of PAI-1 vaccine on survival and a severe systemic symptom (i.e., body weight change) in this model. Mice (n = 5 per group) were immunized three times with the PAI-1 vaccine or KLH vehicle, challenged with mouse-adapted SARS-CoV-2, and monitored for 14 days. Mock-infected mice (n = 5) were not infected with SARS-CoV-2 and were monitored as well. All the mock-infected mice survived as expected, while some of the SARS-CoV-2-infected mice died, but the survival rate after SARS-CoV-2 challenge did not differ between the two infected-mouse groups (*P* = 0.46) (Fig. 5b). In addition, mock-infected mice showed no significant weight changes, whereas all SARS-CoV-2-infected mice showed weight loss. Compared with vehicle-treated mice, PAI-1-vaccinated mice tended to lose weight during the early phase after viral challenge (days 0–4) (*P* = 0.38) but showed no difference in body weight during the late phase (days 5–14) (*P* = 0.92) (Fig. 5c). These results suggest that our PAI-1 vaccine did not attenuate hallmarks of severe COVID-19 (i.e., death and weight loss) in a mouse model of SARS-CoV-2 infection.

### Safety of PAI-1 vaccine

To examine whether PAI-1 vaccine could affect hemostatic parameters, we measured activated partial thromboplastin time (APTT), prothrombin time (PT), bleeding time, and bleeding amount. No significant differences were observed in all the parameters between the vehicle and vaccine group (n = 3 per group) (Supplementary Fig. S1). PAI-1-vaccinated mice also showed no spontaneous hemorrhage. Furthermore, there were no significant differences in serum levels of Cr, UN, ALT, and AST between the two group (Fig. 2b–e). These results suggest that PAI-1 vaccine itself did not show apparent adverse effects and could be a safe treatment option.

## Discussion

Here we developed a peptide vaccine that blocked the antifibrinolytic action of PAI-1 in an effort to prevent thromboinflammation-induced exacerbation of COVID-19. Immunization with our PAI-1 vaccine elicited antibodies that specifically bound to PAI-1. Moreover, the antigen-specific IgG titers were sustained over a long period and ameliorated organ damage, mortality, and impaired fibrinolytic system in LPS-induced sepsis model mice. In a clot lysis assay using healthy human plasma, IgG elicited by our PAI-1 vaccine inhibited the anti-proteolytic activity of PAI-1 against tPA. However, in a mouse model of SARS-CoV-2 infection, our PAI-1 vaccine provided no beneficial effects in terms of decreased body weight loss and mortality.

Although the pathogenic mechanism underlying COVID-19 exacerbation is not well understood, coagulopathy and thrombosis associated with endothelial dysfunction are considered to be key contributors to life-threating conditions, and the available evidence suggests that PAI-1 might play a pivotal role in this pathogenesis^14, 22–24^. The cytokine storm syndrome induced by SARS-CoV-2 can be associated with endotheliopathy and coagulopathy^25, 35^. Interleukin 6 and tumor necrosis factor-alpha both promote the production of PAI-1 in endothelial cells; excess PAI-1 induces the secretion of cytokines and chemokines by binding to toll-like receptor 4 on macrophages, thus forming a positive feedback loop and exacerbating disease progression^22, 25, 36^. In fact, treatment with an anti-interleukin 6 receptor antibody reduces serum PAI-I levels and improves clinical symptomatology in patients with COVID-19^25^. Complement activation may also be involved in the development of thrombotic complications in COVID-19. Patients with moderate and severe COVID-19 had increased plasma C5a levels, which could exert prothrombotic effects by upregulating PAI-1 and various tissue factors as well as by activating platelets^15, 23, 37^. In vitro experiments using human pulmonary microvascular endothelial cells have suggested that SARS-CoV-2 spike protein directly enhances PAI-1 production by inducing endothelial dysfunction^38^. Moreover, PAI-1 is well recognized to be highly related to risk factors for severe COVID-19, including old age, obesity, diabetes, hypertension, cardiovascular disease, and chronic obstructive pulmonary disease^26, 39^. In several studies, the plasma PAI-1 level was correlated with disease severity in patients with COVID-19^25, 29–31^.

Hemorrhagic complications are a main concern regarding therapeutic inhibition of PAI-1. However, in one study, people who were heterozygotic for PAI-1 mutation did not experience prolonged hemorrhagic complications^40^; in fact, heterozygosity was associated with longevity and a healthier metabolic profile. Although homogeneous PAI-1 deficiency is often characterized by prolonged bleeding after injury, trauma, or surgery, this disadvantage can be eased by temporary administration tranexamic acid^41^. Furthermore, in the present study, the vaccination against PAI-1 did not cause any obvious bleeding-related adverse events as well as organ damage. Given these features, PAI-1 appeared to be an attractive target for improving the outcome of patients with COVID-19.

As the vaccine epitope, we selected the amino-acid sequence S367–P372 in the surface-exposed reactive center loop (RCL), which is consistent between humans and mice. PAI-1 is a serpin family member with the common crystal structure consisting of three beta-sheets, several alpha-helices, and the RCL; furthermore, it is synthesized by and secreted from many types of cells, including endothelial cells, adipocytes, liver cells, or macrophages; and finally, it is mainly stored in platelets^42, 43^. Like other serpin family members, PAI-1 inhibits the target proteases t-PA and u-PA^24, 44^. The interaction between the ‘bait’ peptide (R369–M370) bound in the RCL and the target proteases results in the cleavage of the bound, after which the proteases are irreversibly translocated to the opposite site of the PAI-1 module, with the rapid insertion of the amino terminal part of the RCL into beta sheet A^45^. Recently developed anti-PAI-1 monoclonal antibodies (MEDI-579 and AS3288802) to the RCL efficiently block PAI-1 activity and show nephroprotective effects in mouse models of diabetic nephropathy and lupus nephritis^46, 47^. Considering these facts, we considered the RCL as a plausible epitope for our PAI-1 vaccine.

Immunization with PAI-1 vaccine decreased kidney and liver injury and improved fibrinolysis in a mouse model of LPS-induced sepsis (0.5 × LD50). In addition, immunization with our PAI-1 vaccine suppressed LPS-induced elevation in serum levels of Cr, UN, ALT, and AST by 21.4%, 22.5%, 29.2% and 18.1%, respectively. In addition, mice immunized with PAI-1 vaccine showed less thrombosis of small renal arteries, and plasma levels of FDP were increased by 42.4% compared with vehicle-treated mice. In addition, PAI-1 vaccination attenuated mortality in a model of LPS-induced severe sepsis (1.25 × LD50); the survival rate dramatically increased from 16.7% to 75.0%. These results are consistent with previous studies reporting that loss of the PAI gene has a protective effect regarding the toxicity of LPS^48–50^. Therefore, neutralizing the action of PAI-1 has potential to protect mice from severe sepsis. At least part of that mechanism involves suppressing thrombus formation via neutralizing the ability of PAI-1 to inactivate t-PA and u-PA, and the results of our in vitro clot lysis analysis strengthen this hypothesis.

However, contrary to our initial hypothesis, this PAI-1 vaccine did not provide any beneficial effect in a mouse-adapted model of SARS-CoV-2; or even worse, mice in the vaccine group tended to lose more weight during the early phase of disease than those in the vehicle group. Several potential reasons may explain why our PAI-1 vaccine was ineffective in this model. First, PAI-1 may play a negligible role (if any) in the pathogenesis of COVID-19 exacerbation. In the present study, PAI-1 levels were lower in the mice with SARS-CoV-2 infection than with LPS-induced sepsis, although increase in PAI-1 levels in both models was statistically significant and was more than equivalent to that seen in severe COVID-19 patients requiring hospitalization compared to healthy controls^30^; the peak PAI-1 levels were 258.2 ng/mL after LPS injection (0.5 × LD50) and 10.8 ng/mL after mouse-adapted SARS-CoV-2 infection (almost 1.0 × LD50). Indeed, whereas some clinical studies have demonstrated that PAI-1 levels in patients with severe COVID-19 were comparable to those in sepsis patients, other studies did not^25, 51–53^. Whether the PAI-1 level correlates with COVID-19 severity and whether the increased level is a cause or consequence of COVID-19 exacerbations remain under debate^25, 29–31, 51–53^. This apparent discrepancy may reflect the timing of evaluation or the presence or absence of anticoagulant therapy. Moreover, the possibility that enhanced production of PAI-1 during SARS-CoV-2 infection has a beneficial consequence for the host, especially during the early stage, cannot be ruled out. Immunothrombosis, by reducing the spread and survival of some pathogens, can achieve the advantage of intravascular immunity unless it is uncontrolled^14^. PAI-1 itself can exert an antiviral effect on several virus families by directly inhibiting the surface glycoprotein cleavage needed for virion maturation^54^. Plasmin, the levels of which are expected to increase due to the inhibition of PAI-1, is another important cleaving protease that promotes the infectivity of many viruses^55^. In addition, upregulation of PAI-1 can facilitate trafficking of neutrophils into infectious sites and modulate neutrophil viability and apoptosis, resulting in pathogen clearance^56^. In fact, monoclonal antibodies against PAI-1 were not protective in a cecal ligation and puncture-induced sepsis mouse model^57^. In the context of these findings, PAI-1 might contribute to the host defense response against SARS-CoV-2 infection, but because of technical difficulties we were unable to evaluate the effect of our PAI-1 vaccine on viral load. Finally, and perhaps most importantly, our results may reflect a difference in the physiological role of PAI-1 between humans and mice. The plasma PAI-1 level is much lower in wild-type mice compared with humans, and complete PAI-1 deficiency in mice, unlike in humans, leads to only a mild hyperfibrinolytic state, indicating that anti-fibrinolytic action caused by PAI-1 is weak in mice^40, 58–60^. Therefore, extrapolation of the results of our current mouse experiments to humans should be performed with caution.

In the present study, we developed a peptide vaccine targeting PAI-1. This vaccine efficiently attenuated organ injury and microvascular thrombosis and improved survival in LPS-induced sepsis mice, but did not affect outcomes in SARS-CoV-2 infected mice. These results indicate that PAI-1 might not be a major causative factor for exacerbation of COVID-19, at least in a mouse model of SARS-CoV-2 infection.

## Methods

### Animals

We used 6-week-old male C57BL/6J mice (CREA Japan, Tokyo, Japan), which were housed with ad libitum food and water under standard 12:12-h light:dark conditions in accordance with institutional guidelines on animal experimentation at Keio University. Experiments involving SARS-CoV-2 infection were performed in a Biosafety Level 3 facility at the University of Tokyo, with approval from the Ministry of Agriculture, Forestry, and Fisheries of Japan. All animal protocols were approved by the Keio University Institutional Animal Care and Use Committee (approval number 20034) and the Animal Experiment Committee of the Institute of Medical Science, the University of Tokyo (approval number PA21-11) and followed ARRIVE guidelines^61^. Mice were euthanized by cervical dislocation.

### Synthesis of PAI-1 vaccine and immunization protocol

PAI-1 can exhibit three different conformations: the active form, the latent form, and the cleaved form^45, 62^. The active form exposes the RCL, whereas it is fully incorporated into beta-sheet A in the other forms^40^. The RCL of PAI-1 has a crucial role in its inhibitory action on t-PA and u-PA; interaction between the P1–P1′ peptide (amino acids R369-M370) in RCL and the active site of those proteases results in both their inhibition and the inactivation of PAI-1, thereby blocking the degradation of fibrin by preventing the generation of plasmin^42, 44, 45^. In addition, considering various amino acid characteristics including hydrophilicity and electric charge, we selected the sequence SARMAP (GenPept accession no. NP_000593.1; amino acids S367–P372) in the RCL, a sequence that contains the binding site for P1–P1′, as the epitope for the vaccine. The PAI-1 partial peptide was conjugated to KLH (Thermo Fisher Scientific, Waltham, MA, USA) as the carrier protein by using the crosslinker m-maleimidobenzoyl-N-hydroxysuccinimide ester (Eurofins Genomics, Tokyo, Japan). Before administration, we mixed 20 µg of PAI-1–KLH peptide in 100 µL of phosphate-buffered saline (PBS) with an equal volume of Freund’s adjuvant (Sigma–Aldrich Japan, Tokyo, Japan). Complete Freund’s adjuvant was used for the first vaccination; we used incomplete Freund’s adjuvant for the second and third vaccinations. At 6 weeks of age, mice were divided into two groups (vaccine and vehicle groups); they then subcutaneously received 20 µg of either PAI–KLH or KLH peptide with adjuvant at 6, 8, and 10 weeks of age.

### LPS model protocols

As a preliminary investigation, we intraperitoneally administered various doses of LPS (L2630; Sigma-Aldrich) to wild-type mice and determined its 72-h LD50 as 12 mg/kg. Next, to evaluate the effect of LPS administration on the PAI-l level, wild-type mice received intraperitoneal administration of 6 mg/kg LPS (0.5 × LD50) at 13 weeks of age, and blood samples were collected from the retro-orbital plexus before administration and at 6, 12, and 24 h afterward. In the LPS-induced sepsis model to evaluate the effects of PAI-I vaccine on organ injury and thrombus formation, mice immunized with either the vaccine or vehicle received LPS (6 mg/kg IP) at 13 weeks of age (three weeks after the last immunization). Mice were euthanized 24 h after LPS administration, and blood samples and left kidneys were collected. In the LPS-induced severe sepsis model for the survival study, immunized mice received LPS (15 mg/kg IP; 1.25 × LD50) at 13 weeks of age; survival was assessed every 12 h for 72 h.

### SARS-CoV-2 infection model protocols

Mice (age, 20 weeks) were intranasally infected with mouse (C57BL/6J)-adapted SARS-CoV-2 (1 × 10^4^ plaque-forming units), and blood samples were collected before inoculation and at 48 and 96 h afterward, to evaluate changes in the PAI-1 level. In the mouse-adapted model of SARS-CoV-2 to evaluate PAI-1 vaccine effectiveness, survival rate and body weight changes were assessed daily for 14 days in immunized mice that were intranasally infected as described at 16 weeks of age (i.e., 6 weeks after the last immunization). In addition, SARS-CoV-2 untreated mice were observed as mock-infected mice, and their weight changes and survival rates were evaluated daily for 14 days.

### Determination of antibody titers by enzyme-linked immunosorbent assay (ELISA)

To determine serum IgG antibody titers by ELISA, the wells of 96-well microplates (Immulon 1B, Thermo Fisher Scientific) were coated overnight at 4 °C with 1.0 µg of the PAI-1 partial peptide conjugated with bovine serum albumin (BSA) in PBS. The plates were incubated with blocking buffer (PBS containing 0.05% Tween 20 [PBS-T] plus 1% BSA) for 1 h, and subsequently serum samples diluted with blocking buffer were added to each well and incubated for 2 h at room temperature. Plates were then rinsed with PBS-T, goat anti-mouse IgG (SouthernBiotech, Birmingham, AL, USA) was added to each well, and plates were incubated for 1.5 h at room temperature. After plates were rinsed with PBS-T, color reactions generated by using the TMB Microwell peroxidase substrate system (Kirkegaard and Perry Laboratories, Gaithersburg, MD, USA) were evaluated by using a microplate reader (Cytation 5, BioTek, Winooski, VT, USA). The endpoint titer was defined as the reciprocal log_2_ of the highest value in the dilution range of serum samples which gave a wavelength of 450 nm that was 0.1 units greater than that of the negative control^63^.

### Western blot analysis

Western blot analyses were used to determine whether PAI-1 vaccine-elicited antibodies recognize and bind to recombinant full-length PAI-1 protein (A8111, Sigma-Aldrich Japan). KLH, PAI-1 partial peptide conjugated with BSA, and full-length PAI protein underwent sodium dodecyl sulfate polyacrylamide gel electrophoresis and were blotted onto polyvinylidene fluoride membrane (Merck Millipore, Darmstadt, Germany). The blots were incubated with serum samples from mice immunized with either the vaccine or vehicle, followed by incubation with HRP-conjugated anti-mouse IgG secondary antibody (Jackson ImmunoResearch Laboratories, West Grove, PA, USA). The chemiluminescence signal for these blots were visualized by using SuperSignal West Pico Chemiluminescent Substrate (Thermo Fisher Scientific) and detected with an ImageQuant LAS 4000 mini chemiluminescence imager (GE Healthcare Bio-Sciences, Tokyo, Japan).

### Biochemical examination

Serum PAI-1 level was measured by using the Mouse PAI-1 ELISA kit according to the manufacturer’s protocol (ab197752; Abcam, Cambridge, MA, USA). In addition, serum levels of Cr, UN, ALT, and AST, and plasma levels of FDP, APTT, and PT (3.2% sodium citrate at a ratio of 1:9 to whole blood) were measured at a commercial laboratory (Sanritsu Zelkova Laboratory, Tokyo, Japan).

### Histologic study

Mice immunized with either the PAI-1 vaccine or KLH vehicle were intraperitoneally injected LPS; 24 h after LPS injection, the left kidneys were harvested, fixed in 4% paraformaldehyde, embedded in paraffin blocks, and sliced into 4-µm thick sections. To detect renal arterial thrombosis, prepared sections were immunostained by using anti-fibrinogen antibody (ab34269; Abcam) at a commercial laboratory (Morphotechnology, Sapporo, Japan).

### Plasma clot lysis assay

To evaluate the fibrinolytic capacity of antibodies elicited by PAI-1 vaccination, we performed plasma clot lysis assays according to a modified method^64^. Briefly, in each well of a microtiter plate, 60 µL of normal human plasma (George King Bio-Medical, Overland Park, KS, USA) was mixed at room temperature with 40 µL of assay buffer (150 mM NaCl, 20 mM Tris–HCl, 0.05% Tween 20), with or without 6 nM t-PA (monteplase, Eisai, Tokyo, Japan) or both 6 nM t-PA and 9 nM PAI-1. Then, 15 mM CaCl_2_ and 2.5 NIH U/mL human thrombin (T7009; Sigma-Aldrich Japan) were added to all wells. In addition, we used an IgG purification kit (Cosmo Bio, Tokyo, Japan) to extract IgG antibodies from mice immunized with either the vaccine or vehicle; 90 nM IgG was added to wells containing plasma, buffer, t-PA, and PAI-1. Plasma clotting and lysis at 37 °C with shaking were monitored by measuring the absorbance at a wavelength of 405 nm every 5 min until 120 min.

### Tail-bleeding assay

To evaluate the bleeding risk caused by PAI-1 vaccination, we performed a tail-bleeding assay^65^. After the anesthesia with intraperitoneal administration of medetomidine (0.3 mg/kg), midazolam (4.0 mg/kg), and butorphanol (5.0 mg/kg), the tail of mice was amputated at 1 cm from the end with surgical blades. The cut section of the tail was vertically positioned approximately 2 cm below the body horizon and immersed in a 50 mL tube consisting 37 °C saline. Mice were monitored for 20 min to record the total sum of bleeding time within the period. Bleeding amount was determined by measuring the body weight before and after the bleeding test.

### Statistical analysis

Data are expressed as mean ± standard error. Survival was visualized according to the Kaplan–Meier method and differences in survival were evaluated by using log-rank tests. Body weight changes were analyzed by using linear mixed-effects models with both random intercept and random slope for the early (days 0–4) and late (days 5–14) phases, respectively. Student’s *t* test was used to compare between groups (i.e., mice immunized with the PAI-1 vaccine vs KLH vehicle in the LPS-induced sepsis model and mouse-adapted model of SARS-CoV-2). For serum PAI-1 levels, pre-challenge and first post-challenge values were compared. All analyses were performed by using SPSS version 27.0 (IBM, Armonk, NY, USA), and figures were created by using Prism 9 (GraphPad Software, San Diego, CA, USA). A *P* value < 0.05 was considered statistically significant.

## Supplementary Information

Supplementary Figure S1.

Supplementary Information 2.

## Data Availability

All data are available in the main text. Any additional information can be obtained from the corresponding author upon reasonable request.
