# Supplementary material for: Plasminogen activator inhibitor 1 is not a major causative factor for exacerbation in a mouse model of SARS-CoV-2 infection
Source: Sci Rep. 2023 Feb 22;13:3103. doi: 10.1038/s41598-023-30305-8 (PMC9944779; doi:10.1038/s41598-023-30305-8)

Supplementary materials

Plasminogen activator inhibitor 1 is not a major causative factor for exacerbation in a mouse model of SARS-CoV-2 infection

Takashin Nakayama , Tatsuhiko Azegami , Maki Kiso, Masaki Imai, Ryuta Uraki, Kaori Hayashi, Akihito Hishikawa, Norifumi Yoshimoto, Ran Nakamichi, Erina Sugita-Nishimura, Eriko Yoshida-Hama, Yoshihiro Kawaoka & Hiroshi Itoh

Supplementary Figure S1. Effects of PAI-1 vaccine on hemostatic parameters. Mice (n = 3 per group) were immunized with the PAI-1 vaccine or KLH vehicle. (**a, b**) Plasma samples were obtained at 13 weeks of age, and activated partial thromboplastin time (APTT) and prothrombin time (PT) were measured. (**c, d**) Tail-bleeding assay was performed at 16 weeks of age. Data are expressed as mean ± SEM (error bars). Significant differences in each parameter between the vaccine and vehicle groups were determined by using Student's *t*-test.

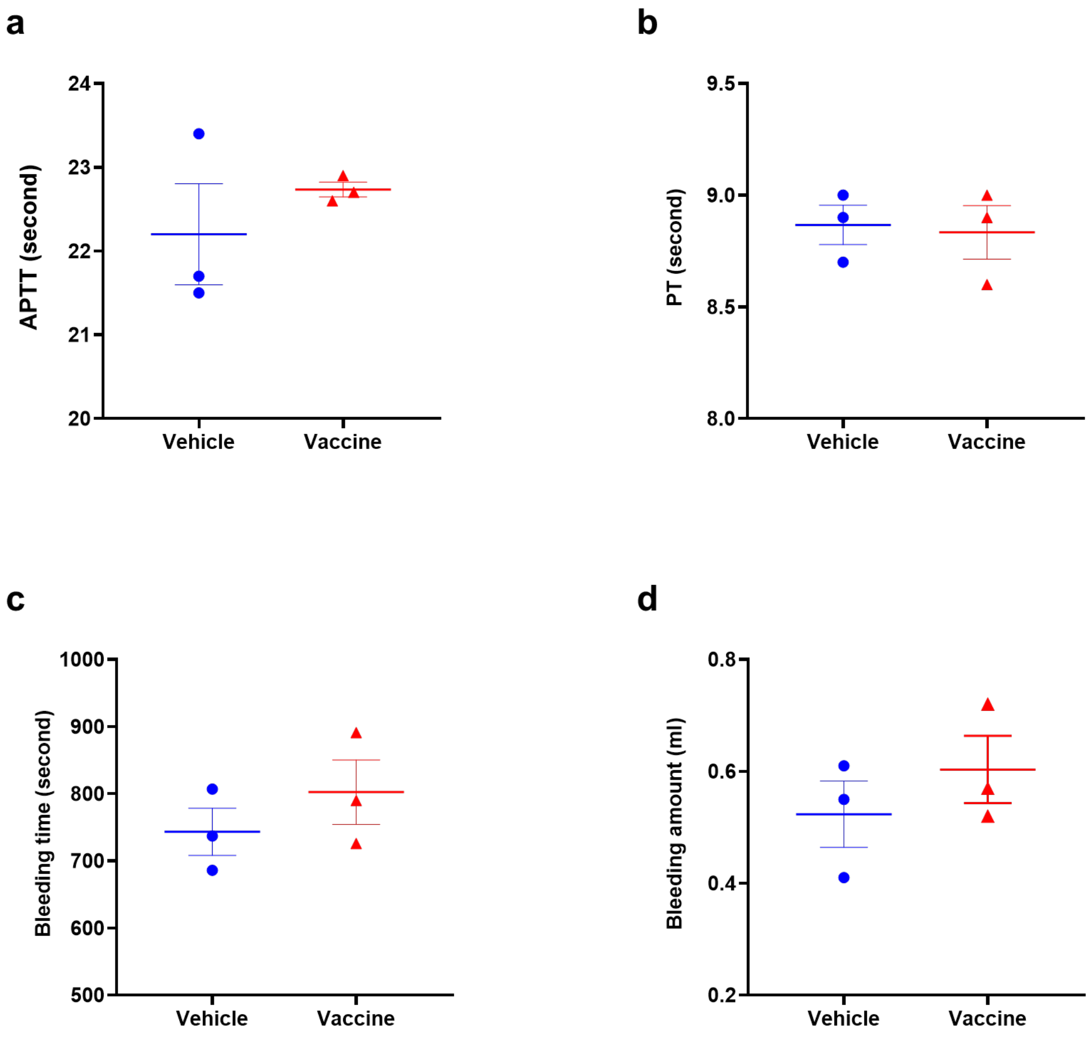

Supplement: Supplementary file 1 — Supplementary Figure S1. [file 41598_2023_30305_MOESM1_ESM.pdf]
