# Supplementary figures and images for: Plasminogen activator inhibitor 1 is not a major causative factor for exacerbation in a mouse model of SARS-CoV-2 infection
Source: Sci Rep. 2023 Feb 22;13:3103. doi: 10.1038/s41598-023-30305-8 (PMC9944779; doi:10.1038/s41598-023-30305-8)

**Vehicle serum**

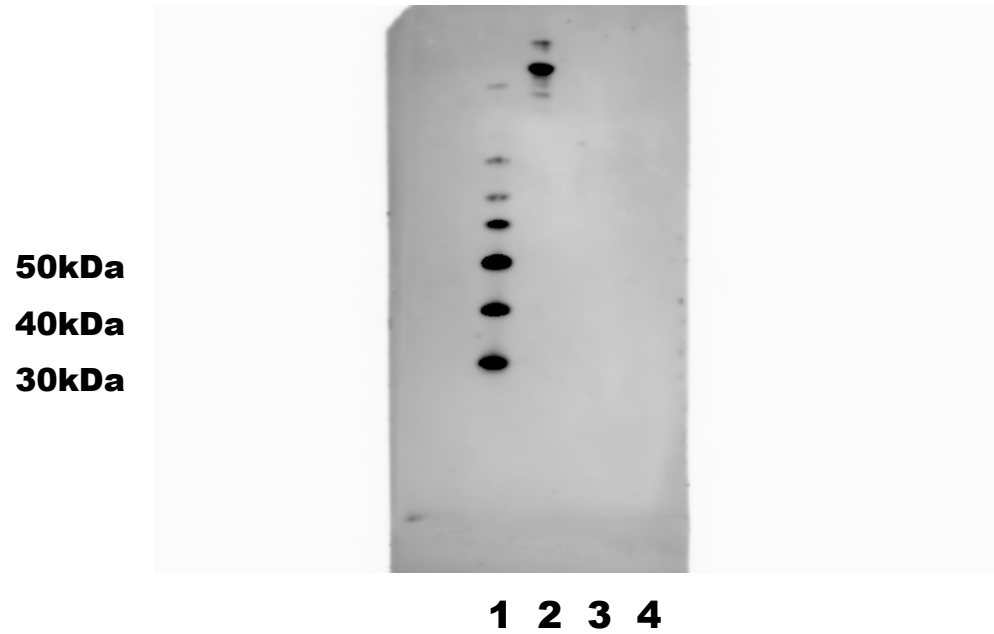

**Vaccine serum**

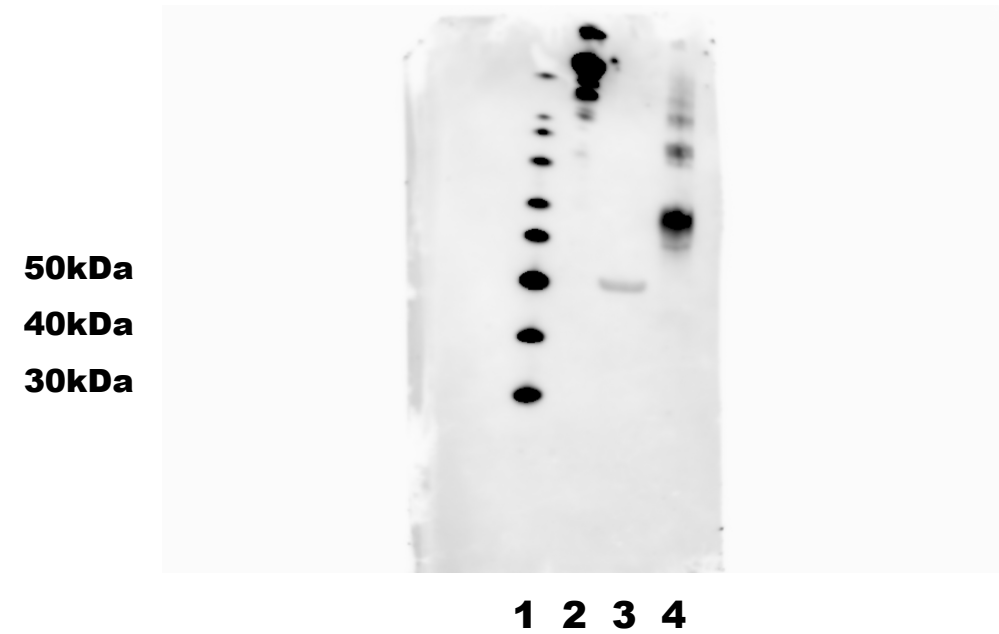

**1: Marker, 2: KLH, 3: recombinant PAI-1 whole protein, 4: PAI-1(366-372)-BSA**

Supplement: Supplementary file 2 — Supplementary Information 2. [file 41598_2023_30305_MOESM2_ESM.pdf]
